# Antiphospholipid syndrome and valvular heart disease, a complex scenario of thrombotic events, a case report

**DOI:** 10.1186/s13019-020-01330-9

**Published:** 2020-09-29

**Authors:** Santiago A. Endara, Gerardo A. Dávalos, Christian H. Fierro, Vladimir E. Ullauri, Gabriel A. Molina

**Affiliations:** 1 Department of Surgery Division of Cardiothoracic Surgery, Hospital Metropolitano, Av. Mariana de Jesús Oe7/47 y Conclina, Edificio Diagnóstico 2000 tercer piso 3/3, Quito, Ecuador; 2 Department of Internal Medicine Division of Cardiology, Hospital Metropolitano, Quito, Ecuador; 3 Colegio de Ciencias de la Salud, Universidad San Francisco de Quito (USFQ), Quito, Ecuador

**Keywords:** Antiphospholipid syndrome, Thrombus, Coronaries arteries, Aortic valve replacement

## Abstract

**Background:**

Antiphospholipid syndrome (APS) is a rare coagulation disorder associated with thrombotic events, myocardial infarction, and valvular heart disease. During valvular replacement surgery, the high risk of thrombosis combined with the operative risks in these specific groups of patients poses a challenge to the medical team.

**Case presentation:**

We present a case of a female patient with APS and mixed aortic valve disease. During surgery, she suddenly developed complete cardiac arrest. Three months later, after she recovered, and while she was still on close follow up, a thrombotic event caused myocardial infarction. After prompt and precise treatment, the patient successfully recovered; one year after surgery patient is doing well.

**Conclusion:**

Adequate surgical technique along with optimal anticoagulation strategies and long term follow up are of paramount importance to ensure an uneventful recovery. A multidisciplinary team is required to manage these complex scenarios and high-risk patients.

## Background

Antiphospholipid antibodies are a heterogeneous group of autoantibodies that have clear associations with thrombosis [1]. Anticoagulation is a vital part of APS treatment as thromboembolic events can cause severe morbidity and mortality. When APS damage a heart valve, surgery becomes a challenging situation [1, 2]. Perioperative treatment and surgical planning become critical steps as the medical team must be adequately qualified to face possible complications and give the necessary long-term treatment and follow up [1, 3].

This work has been reported in line with the CARE criteria [4].

## Case presentation

**Fig. 1:**
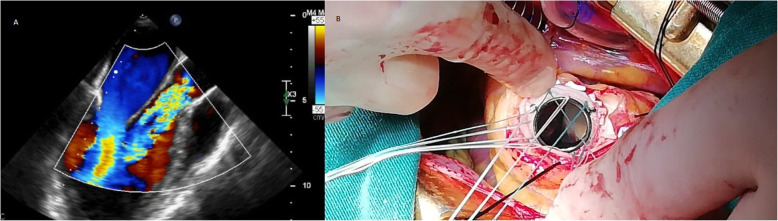
**a** Transthoracic echocardiogram revealing a maximum aortic velocity of 5.36 m/s. **b** A 17-mm prosthetic aortic valve is placed

**Fig. 2:**
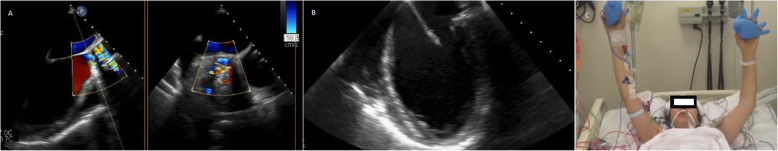
**a** TEE revealing the prosthetic valve without any problems. **b** TEE confirming regional wall motion abnormalities in both ventricles. **c** Patient on postoperative day one, without neurological defects

**Fig. 3:**
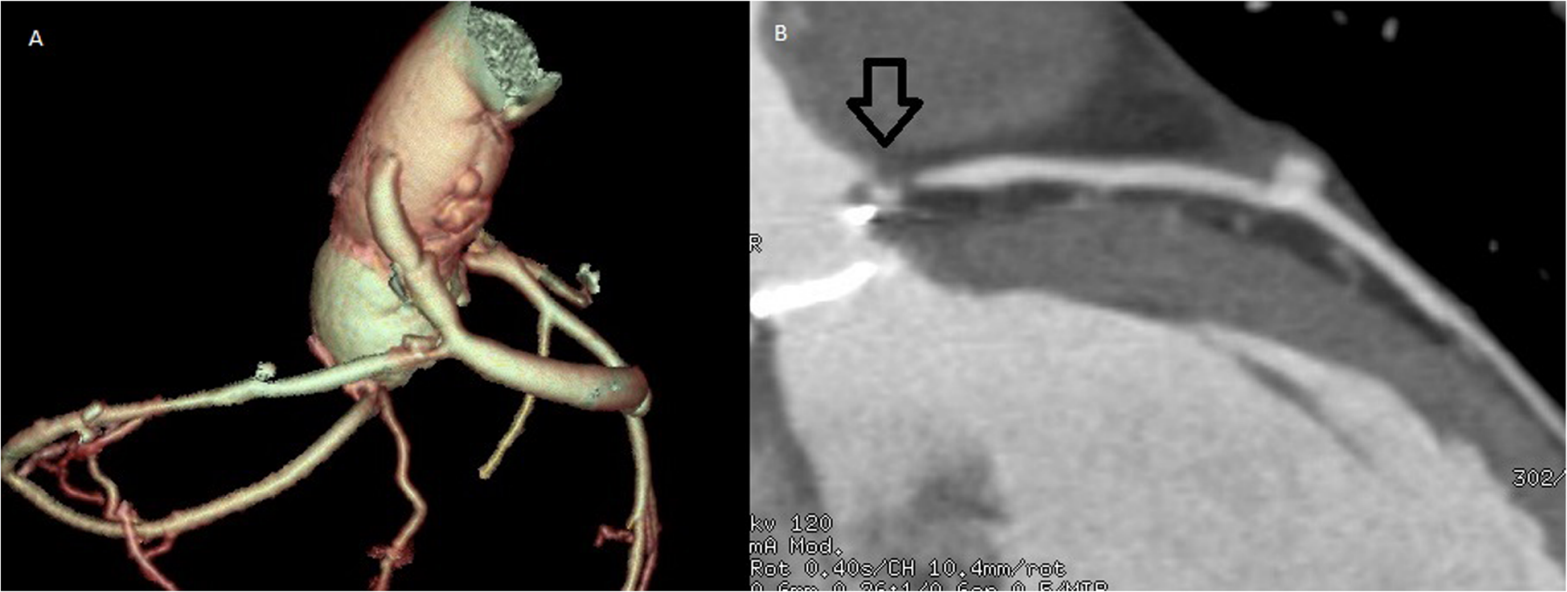
**a** CT-coronary angiogram revealing total ostial obstruction of both coronary arteries. **b** CT-coronary angiogram revealing obstruction on the left coronary artery

Patient is a petite 35-year-old vegan female with past medical history of systemic lupus erythematosus (SLE), antiphospholipid syndrome (APS), and deep vein thrombosis complicated with pulmonary embolism for which she received rivaroxaban. On routine medical examination, while she was completely asymptomatic, a holosystolic murmur was detected, thus, echocardiography was requested. It revealed a dilated left ventricle with mild hypertrophy and an ejection fraction of 58%. Her aortic leaflets were thickened with limited mobility, her maximum aortic velocity was 4.36 m/s, and the mean gradient was 45 mmHg. A severe regurgitant flow was at the aortic valve confirming severe aortic insufficiency (AI) and moderate aortic stenosis (AS). Mixed aortic valve disease was diagnosed and due to her clinical background, surgery was decided, nonetheless anxiety and fear caused her to refuse surgery. One year passed and although she remained asymptomatic, her condition worsened, maximum aortic velocity was 5.36 m/s, the mean gradient was 60 mmHg, and aortic valve area was 0.9 cm2. Also noted was an increase in the left ventricular diastolic diameter due to severe aortic Insufficiency (Fig. 1a). After great help from her family and psychology treatment, surgery was accepted. Sternotomy and cardiopulmonary bypass (CPB) were completed after a dose of 300 U/kg was administered and the ACT (activated clotting time) was over 480 s, the ACT was monitored every 30 min. Cardiac arrest was achieved via antegrade and retrograde cardioplegia, the temperature was kept at 35 C and cold cardioplegia was administrated every 10 min. Aortotomy was done. The aortic valve was visualized, revealing fibrous and stenotic aortic leaflets. The leaflets were excised, and a 17-mm prosthetic aortic valve was placed (St. Jude Medical, Inc., St. Paul, Minn) after we measured her aortic annulus diameter (18 mm) (Fig. 1b). Both coronary vessels were identified and spared during the valve replacement. Once the valve was sewn and placed, the aortotomy was closed with a 5–0 polypropylene suture in a two-layer fashion. Before removing the aortic cross-clamp warm blood retrograde cardioplegia was given. The cross-clamp was removed, and the heart reverted in sinus rhythm. With a transesophageal echocardiogram (TEE) the prosthetic valve was evaluated and didn’t exhibit any problems (Fig. 2a). The patient was weaned off CPB using a 50 mg test dose of protamine. With no hemodynamic instability from this dose, the rest of the protamine was given slowly over 15 min. While finishing the procedure as the sternal wires were placed, the patient suddenly suffered from severe hypotension, bradycardia, and sudden cardiac arrest. The sternal wires were removed, and cardiopulmonary resuscitation was started immediately with open cardiac massage and repeated direct defibrillation with 20 joules, heparin was given, aortic and atrial cannulation was done during the cardiac massage and the patient was placed again under CPB. Since the heart had multiple zones of hypokinesia and akinesia in both ventricles. ECG at that time registered pulseless electrical activity. Using TEE, we confirmed regional wall motion abnormalities in both ventricles (Fig. 2b and Supplementary Video (1)). The aortic prosthesis had a normal function and we suspected a coronary perfusion problem. Since it was difficult to locate the exact coronary lesion, we decided to perform an immediate coronary artery bypass graft (CABG) surgery. We used saphenous vein grafts to the left anterior descending and the right coronary arteries. The myocardium showed signs of definite clinical improvement following revascularization. Inotropic and vasoactive agents were partially weaned off along with CPB, the patient was transferred to the intensive care unit for close monitoring. Neurological complications were suspected due to the cardiac arrest, nonetheless, the patient satisfactorily recovered from the procedure without any neurological deficits. Her postoperative course was uneventful despite developing posterior wall MI (myocardial infarction) with preserved ejection fraction, she was fully aware on her first postoperative day and never showed signs of neurological complications (Fig. 2c and Supplementary Video (2)). Anticoagulation was managed with low-molecular-weight-heparin and warfarin was continued until the international normalized ratio (INR) achieved a value ranging between 2.5 and 3.5. She was discharged on her eighth postoperative day. Anticoagulation (Unfractionated heparin and a vitamin-K antagonist) was difficult due to her diet, but she remained on close follow-up controls, with hematologist. A Computed tomography (CT)-coronary angiogram at that time revealed total ostial obstruction of both coronary arteries trunks, and adequate flow through both saphenous vein grafts. On follow-ups, patient was doing well (Fig. 3a, b).

**Fig. 4:**
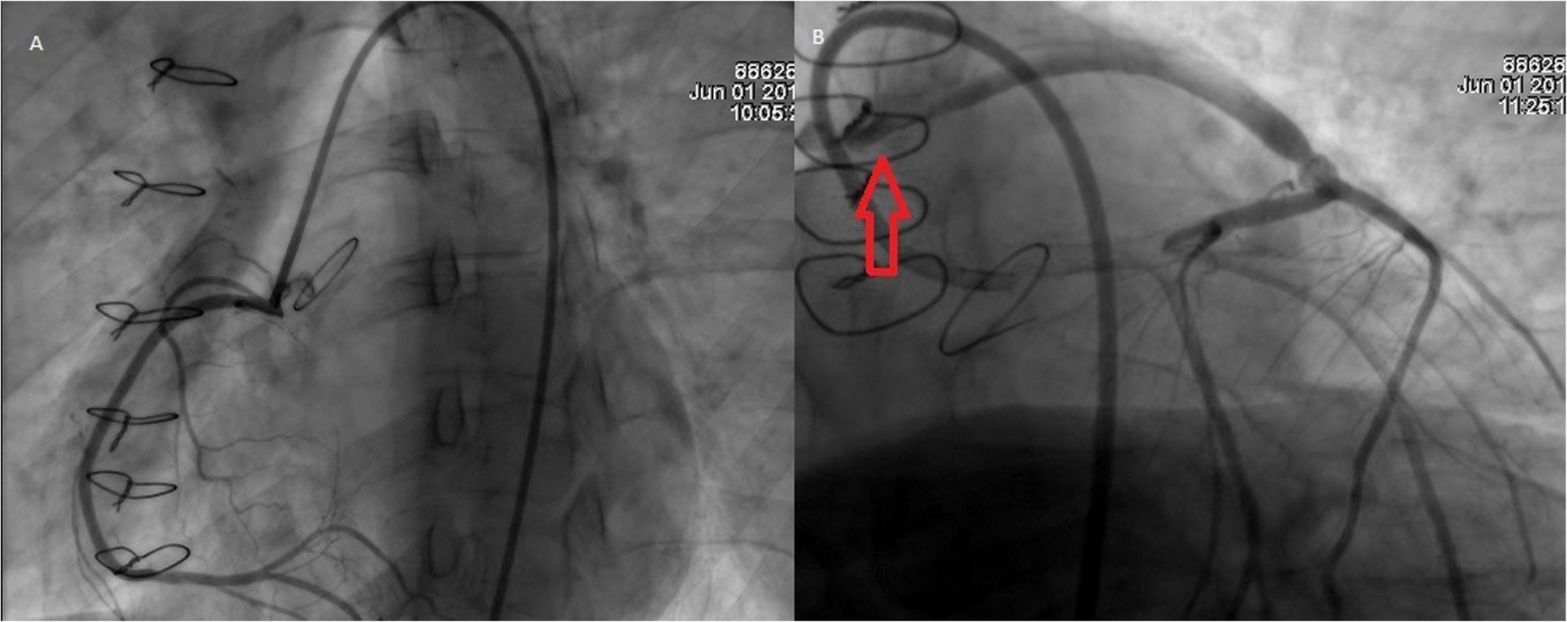
**a** Coronariography, right coronary artery angiogram, showing a patent ostium. **b** Coronariography, Severe thrombotic obstruction of the proximal vein graft to the LAD

**Fig. 5:**
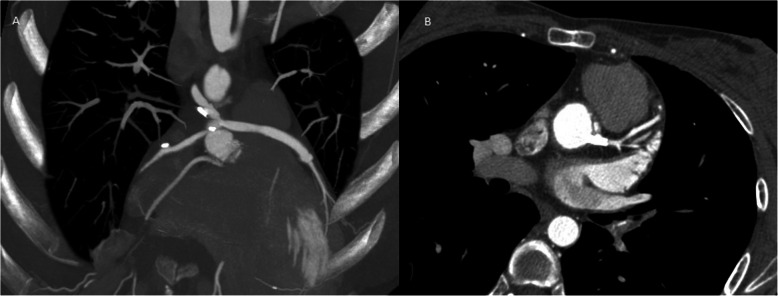
**a** CT-coronary angiogram, three months after revascularization showing patent stent

Nonetheless 3 months after surgery she suddenly experienced acute chest pain, shortness of breath, nausea, and fatigue thus was brought immediately to the emergency room. On clinical examination, a tachycardic and diaphoretic patient was encountered. An electrocardiogram (EKG) detected an anterior myocardial infarction with ST-elevation, along with troponin values above 130 pg/L and INR of 2.27. Femoral access was achieved, and percutaneous coronary intervention was decided. On coronariography, (Fig. 4a, b) the right native coronary artery appeared normal and to our surprise it recanalized spontaneously. However, the left coronary artery occlusion persisted and there was a severe thrombotic stenosis of the left saphenous vein bypass. The right saphenous vein bypass to the right coronary artery was also totally occluded. After successful intraortic balloon pump (IABP) insertion a stent was placed on the saphenous vein graft achieving adequate blood flow improving the patient’s condition. Transthoracic echocardiogram revealed anterolateral hypokinesia of the left ventricle, with an ejection fraction of 45%. After this procedure, the patient fully recovered and had no cardiac symptoms. She was discharged under close anticoagulation surveillance. Three months later and due to the fact that the patient had experienced early graft failure and her young age, it was decided to perform percutaneous revascularization of the main left coronary trunk which was performed successfully (Fig. 5a) with intravascular ultrasound guidance. She completely recovered from the procedure and 1 year after surgery on regular follow-ups, the patient is doing well without any complications.

## Discussion and conclusions

Antiphospholipid syndrome (APS) is an immune-mediated acquired thrombophilia characterized by venous and arterial thrombosis, fetal losses, thrombocytopenia and the presence of antiphospholipid antibodies, in some patients, this syndrome can be associated with systemic lupus erythematosus (SLE) [1, 2]. As it occurred in our case. APS is found in up to 5% of the general population and up to 30% of patients with SLE. One of the many clinical manifestations of APS is valvular heart disease [1, 3]. Heart valve disease appears in 14,3% of patients with APS and is defined by the presence of irregular nodules or vegetations, and moderate to severe valve dysfunction in the absence of a history of rheumatic fever or infective endocarditis [2, 3]. APS tends to damage the left heart valves with predilection on the mitral valve. Although the pathophysiology is not yet fully understood, it is believed that APS, causes immune complexes that injure the valvular endothelium causing fibrotic changes on the valve [1, 2]. Heart valve disease in the setting of APS is related to a 10-fold increase in the risk of stroke [3, 5, 6]. The damage to the valve is regularly mild, and the likelihood of developing heart valve disease is around 8%. When it occurs, valve replacement surgery is needed in up to 6% of these patients [3, 5, 7]. In asymptomatic patients with AS, surgery is recommended when the left ventricular ejection fraction is less than 50% or when exercise stress tests are abnormal and should be considered when the mean pressure gradient is beyond 60 mmHg, the aortic jet velocity is above 5 m/s, there is evidence of a left ventricular hypertrophy, elevated B-type natriuretic peptide and reduced global left ventricular strain in low-risk patients [8, 9]. Nonetheless, our patient had APS that made her situation even more complicated, not only because of the increased surgical risk but because of her perioperative management. Even so, early surgical aortic-valve replacement should be considered in these patients as a significantly lower risk of operative mortality is found than conservative management among asymptomatic patients with very severe aortic stenosis [10]. When valve replacement is necessary the morbidity and mortality for these patients are higher (50 and 22%) compared to the mortality in non-APS patients 2% [1, 8, 9]. This may be accounted for by several factors including, renal insufficiency, anemia, and thromboembolism. Perioperative thrombosis caused by antiphospholipid antibodies during the procedure can be catastrophic and should always be considered and prevented [5, 9, 11].

Acute coronary obstruction during heart surgery is a very rare but real problem occurring in at least 0.14% of patients. Regretfully when it occurs it is usually fatal [12, 13]. There are few cases reports in the literature of this dreadful condition. Obstruction from the aortotomy sutures, post-traumatic thrombosis due to the aortic retractor, embolism from debris, and surgical glues have been found as the cause of coronary obstruction [7, 9]. Nonetheless, after an extensive search in the literature, we haven’t found a report of this phenomenon in the setting of an APS patient with an aortic valve replacement surgery. We suggest that the mechanism of obstruction was due to an acute local thrombus that formed around the coronary ostium that ultimately caused the coronary obstruction and finally the cardiac arrest. Careful surgical techniques are essential, and care must be taken by some basic precautions and intraoperative checks on ostial integrity [11, 14]. If after with all these precautions, coronary compromise is detected, many options are available including coronary stenting, device removal, re-replacement with a smaller valve size, coronary graft, and percutaneous coronary intervention [1, 15, 16]. In our patient, thrombus probably secondary to APS occluded both coronaries’ arteries leading to a nearly fatal complication, thankfully the CABG worked, and the patient survived this event.

APS is also associated with acute myocardial infarction (AMI). This thromboembolic state caused by, endothelial cell activation, direct inhibition of the activated protein C pathway, abnormalities in platelet function and complement activation can predispose to cardiovascular events [6, 17]. Up to 5.5% of cases with AMI in young individuals are secondary to APS. When a stent is used in a patient with APS, the antiphospholipid antibody on intima in the stent promotes atherosclerosis, restenosis as well as vein graft disease [17–19].

We suspected that the coronary stenosis found in our patient was due to the proliferation of the intima and the formation of thrombus on the coronaries arteries, as the patient received high-dose statins, antiplatelet and anticoagulant therapy.

The natural history of thrombus will depend on the rate of absorption, blood flow around the obstruction, degree of organization, anticoagulation, and fibrinolytic mechanisms. There have been several reports of spontaneous revascularization of arterial vessels (coronaries, renal and popliteal arteries) [10, 20]. It is believed that many numbers of endothelial stimuli, including ischemia, may enhance fibrinolysis, which in turn can resolve the thrombus [21, 22]. We believe that in the setting of a patient with APS and several thrombotic events, many emboli could have been formed that resolved spontaneously, nonetheless the ones that were formed around the coronary arteries completely change her prognosis.

Patients with APS have higher long-term rates of adverse cardiac events when compared to both the general population that is why are that an early and aggressive approach to the treatment of patients with APS is justified. Balancing the risks of thrombosis and bleeding is needed to develop optimal individualized therapy for patients with APS [23, 24].

## Conclusion

APS is a complex disease that must be managed by a multidisciplinary team of surgeons, physicians, and hematologists, given the severe spectrum of cardiac complications that this disease can exhibit precise surgical skills, a close follow-up and precise anticoagulant treatment are crucial to improve the patient’s outcome when these rare scenarios arise.

### Patient perspective

At first, the patient was unsure about her treatment, her main concerns were how long it would last, whether it would hurt and whether she could be “normal” again. However, after the surgery and discharge, she gained more confidence and as she was feeling well, she partially ignored her diet and his anticoagulant therapy. After her last cardiac event, she realized how important her therapy is, and is on close follow-ups.

## Supplementary information

**Additional file 1: Supplementary Video (1):** TEE video revealing open heart compressions.

**Additional file 2: Supplementary Video (2):** Patient on UCI, without neurological defects.

## Data Availability

Not Applicable.

## Abbreviations

APS: Antiphospholipid syndrome
SLE: Systemic lupus erythematosus
AI: Aortic insufficiency
AS: Aortic stenosis
CPB: Cardiopulmonary bypass
TEE: Transesophageal echocardiogram
CABG: Coronary artery bypass graft
INR: International normalized ratio
CT: Computed tomography
IABP: Intraortic balloon pump
